# Picophytoplankton dynamics in a large temperate estuary and impacts of extreme storm events

**DOI:** 10.1038/s41598-020-79157-6

**Published:** 2020-12-16

**Authors:** Ryan W. Paerl, Rebecca E. Venezia, Joel J. Sanchez, Hans W. Paerl

**Affiliations:** 1 Department of Marine Earth and Atmospheric Sciences, North Carolina State University, Raleigh, NC 27695-8208 USA; 2 Institute of Marine Sciences, University of North Carolina At Chapel Hill, Morehead City, NC 28557 USA

**Keywords:** Microbiology, Ocean sciences, Microbial ecology, Natural hazards, Hydrology

## Abstract

Picophytoplankton (PicoP) are increasingly recognized as significant contributors to primary productivity and phytoplankton biomass in coastal and estuarine systems. Remarkably though, PicoP composition is unknown or not well-resolved in several large estuaries including the semi-lagoonal Neuse River Estuary (NRE), a tributary of the second largest estuary-system in the lower USA, the Pamlico-Albemarle Sound. The NRE is impacted by extreme weather events, including recent increases in precipitation and flooding associated with tropical cyclones. Here we examined the impacts of moderate to extreme (Hurricane Florence, September 2018) precipitation events on NRE PicoP abundances and composition using flow cytometry, over a 1.5 year period. Phycocyanin-rich *Synechococcus*-like cells were the most dominant PicoP, reaching ~ 10^6^ cells mL^−1^, which highlights their importance as key primary producers in this relatively long residence-time estuary. Ephemeral “blooms” of picoeukaryotic phytoplankton (PEUK) during spring and after spikes in river flow were also detected, making PEUK periodically major contributors to PicoP biomass (up to ~ 80%). About half of the variation in PicoP abundance was explained by measured environmental variables. Temperature explained the most variation (24.5%). Change in total dissolved nitrogen concentration, an indication of increased river discharge, explained the second-most variation in PicoP abundance (15.9%). The short-term impacts of extreme river discharge from Hurricane Florence were particularly evident as PicoP biomass was reduced by ~ 100-fold for more than 2 weeks. We conclude that precipitation is a highly influential factor on estuarine PicoP biomass and composition, and show how ‘wetter’ future climate conditions will have ecosystem impacts down to the smallest of phytoplankton.

## Introduction

Picophytoplankton (PicoP) are phytoplankton < 2–3 µm in diameter that are present in nearly all aquatic systems^1–3^. PicoP are widely considered key contributors to primary productivity (PP) and phytoplankton biomass (PB) in oligotrophic lakes and the pelagic ocean^3,4^. However, mounting evidence points to PicoP as significant contributors to PP and PB in more productive meso- and eutrophic systems as well—including highly productive estuaries. In several large estuaries PicoP account for > 25% of average PP and PB, and at times 100% during summer months^5–8^.

Based on cell size and pigment content, PicoP are partitioned into picocyanobacteria (Picocyanos) and picoeukaryotic phytoplankton (PEUK) by microscopy and flow cytometry^9–11^. The morphotypes present in estuaries vary in terms of absolute and relative abundances^12,13^. Picocyanos are composed of unicellular phycoerythrin (PE)-rich and phycocyanin (PC)-rich cells frequently attributed to the genus *Synechococcus*, but may include other genera, e.g. *Cyanobium*, *Synechocystis*^14,15^. *Synechococcus*-like PC-rich cells (PC-SYN) often numerically dominate waters < 25 salinity, while *Synechococcus*-like PE-rich cells (PE-SYN) tend to dominate waters of salinity > 25^8,16,17^. This salinity dichotomy is evident in many estuaries, but with some exceptions, e.g. blooms of PC-rich *Synechococcus* in saline to hypersaline Florida Bay^18^. PEUK are also common in estuaries^3,19^ and include Chlorophytes, Pelageophytes, Haptophytes, and small diatoms^20^. In some estuarine and coastal systems PEUK can equal or overtake Picocyanos in abundance as well as biomass^21–27^.

Data on PicoP morphotype abundances, biomass and genetic diversity are available from various estuaries^3,12,13,28^ including Chesapeake Bay, the largest mainland US estuary^8,29^; notably though, there is less equivalent data from the second largest US estuary, the Pamlico-Albemarle Sound System (PASS) North Carolina (NC). The Neuse River Estuary (NRE) is a tributary of the greater PASS. It is a temperate system with well-documented spatial gradients in salinity, inorganic and organic nutrients, and turbidity extending from near New Bern, NC to the river mouth broadening to the Pamlico Sound^30–32^. PicoP significantly contribute to PP and PB in the NRE, on avg. ~ 40% and > 70% during summer periods, and are desirable prey for nano- and micro-zooplankton^7,33^. *Synechococcus*-like cells are thought to be the most prevalent NRE PicoP based on microscopy^7^. However, PicoP morphotypes have not been specifically examined and contributions of specific PicoP morphotypes to biomass and carbon fixation are not well constrained. The prevalence of certain PicoP cell types can potentially impact ecosystem trophodynamics, as some strains are resistant to grazing and contribute less to sustaining upper levels of the food web^34,35^, as well as health of co-occurring phytoplankton^36^ and behavior of co-occurring fauna^37,38^.

From meteorological, climatological, and ecological perspectives, the NRE is located along a section of the US coastline that is increasingly influenced by extreme weather, including 38 tropical storms and hurricanes since 1996 that have led to record-setting precipitation and flooding events^32,39,40^. The negative impacts of extreme weather on coastal ecosystems is a global concern and important to resolve in order to predict biogeochemical and trophic consequences of future environmental change. Over two decades of water quality and biological data from the NRE highlights extreme storms as the cause of elevated dissolved organic carbon flux as well as shifts in phytoplankton composition and biomass^32,41^. The impacts of moderate as well as extreme storms on PicoP are largely unknown in the NRE. Significant reductions in the diagnostic photopigment zeaxanthin in the NRE occur after high precipitation tropical storms and are attributed to reductions in cyanobacteria, likely Picocyanos^32,42^. Notably though, PEUK strains can also produce zeaxanthin^43^ and Picocyanos and PEUK often co-occur^3,12,44^, all of which makes it challenging to equate zeaxanthin declines with declines in specific PicoP.

In temperate and tropical estuaries, elevated river inputs can have diverse effects on PicoP, including shifts of the *Synechococcus* community towards PC-rich cells and freshwater populations^8,13,16,17^, reductions in *Synechococcus* abundance^13,16^, and changes in PEUK abundance^44,45^. Increased river flow in the NRE tends to reduce cyanobacteria while promoting Chlorophytes and Cryptophytes based on diagnostic photopigments^46^. Putatively, PicoP representatives account for changes in these groups, e.g. *Synechococcus*-like cells^7^.

To elucidate PicoP composition and abundance in the NRE, we examined PicoP morphotypes using flow cytometry (FCM) along the fresh to polyhaline NRE continuum over a hydrologically variable 1.5 year period. Frequent storm events in the region offered the opportunity to specifically assess the impact of moderate to extreme storms on estuarine PicoP as well. One extreme event, Hurricane Florence, passed over eastern NC and Neuse River Basin during this study—delivering record rainfall, > 86 cm over ~ 3 days in areas, and causing extensive flooding within the Neuse River watershed^40,47^.

## Materials and methods

### Water sample collection and hydrologic measurements

**Figure 1:**
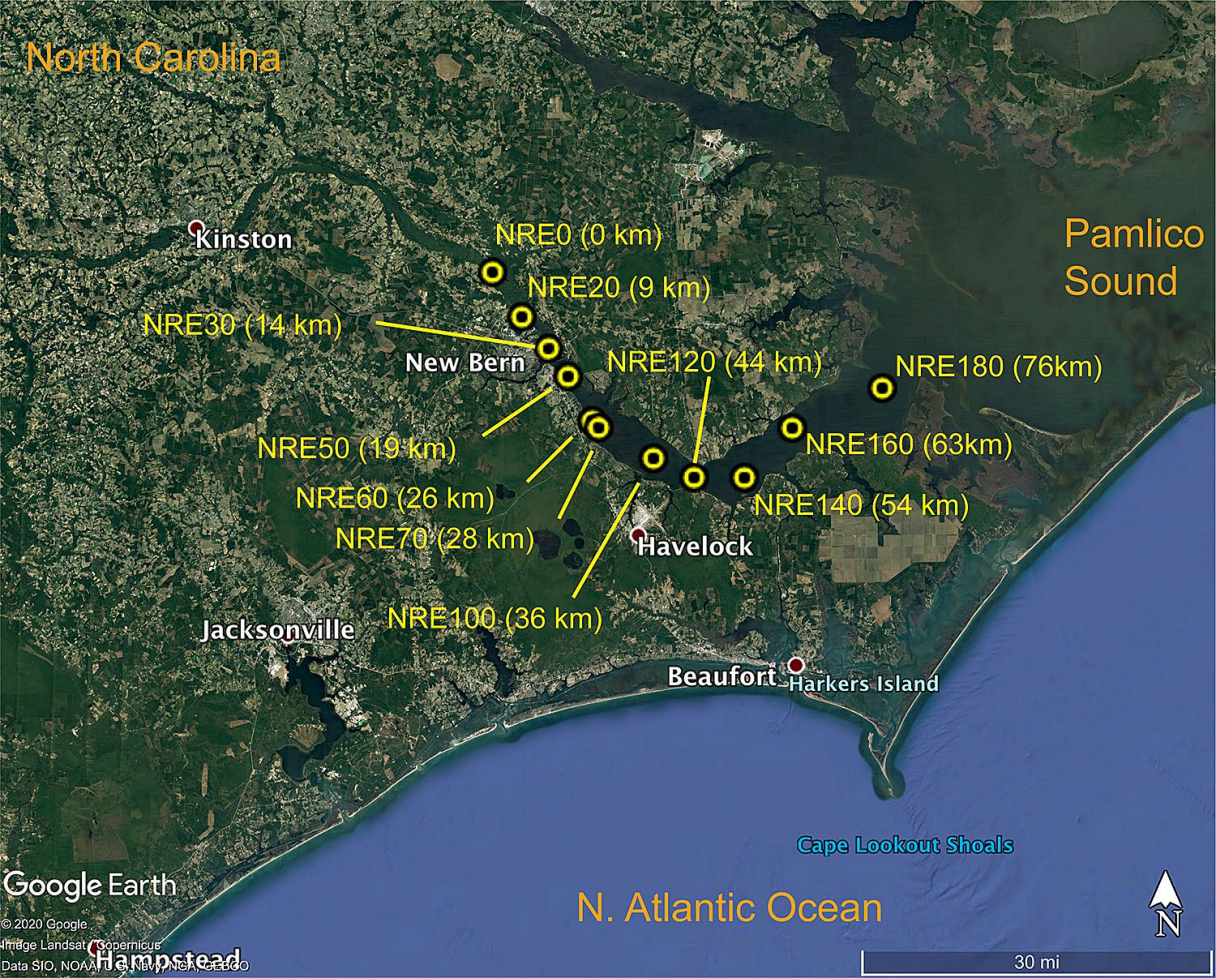
Neuse River Estuary (NRE) stations sampled in this study. The distance down river from station NRE0 is provided in parenthesis. NRE stations are those established as part of the MODMON monitoring program. The underlying map image was retrieved using Google Maps Pro version 7.3.3.7786 (https://www.google.com/earth/versions/#download-pro).

Near surface water samples were collected via non-destructive diaphragm pump at 11 stations on the NRE from riverine station 0 to the most saline river mouth station 180 as part of the Neuse River Estuary Modeling and Monitoring Project (MODMON) (http://paerllab.web.unc.edu/projects/modmon/) (Fig. 1). Samples were collected bi-monthly to monthly from July 2017 to December 2018 following the MODMON sampling scheme^32^. The surface water was kept on ice in dark coolers and transported to NC State University, Raleigh, NC for processing the next day. Hydrologic data were obtained by MODMON protocols^7^ (Supplementary Datatable 1). Neuse River flow data from USGS gauge 02091814 near Ft. Barnwell were retrieved from the USGS National Water Information System Web Interface: https://waterdata.usgs.gov/nwis.

### Sampling for FCM and FCM-based PicoP enumeration

Water samples for FCM analysis were fixed with glutaraldehyde at 0.25% final conc. and placed in the dark for ≥ 15 min before storage at − 80 °C. Thawed FCM samples were analyzed using a dual laser (488 nm, 600 nm) Guava EasyCyte HT (Millipore) flow cytometer and GuavaSoft (Millipore) by triggering event counts off the blue laser (red fluorescence; typical of Chl *a* possessing cells). Initial tests were completed to analyze the background noise and potential coincidence that can impact counting. Following initial tests, samples were diluted 1:20 with 0.2 µm filtered deionized (DI) water. Distinct PicoP morphotypes were identified and enumerated based on autofluorescence from blue and red laser excitation^12^. For enumeration of PE-SYN cells, secondary gating based on forward scatter (FSC) was used to exclude events due to particles associated with instrument cleaning solution which was observed in DI blanks (Supplementary Fig. S1). Triplicate samples, in rare cases duplicate/single samples due to sample loss, were run to obtain mean and standard deviation cell count data. The limit of quantification (LOQ) for FCM analysis was 1.11 × 10^3^ cells mL^−1^ based on detection of a single cell from the average sample volume processed from summer and winter samples. Samples with undetectable levels of PicoP morphotypes were excluded from statistical analysis. Spherical reference beads of known diameter (0.5–5.11 µm; Spherotech) were used to obtain PicoP cell diameter estimates for morphotypes based on FSC, using an empirically-determined linear relationship between FSC and bead diameter (Supplementary Fig. S2). Assuming a spherical shape for all PicoP, biovolume was calculated as well as carbon biomass units per PicoP cell using the conversion factor of 237 fg C µm^−3^^24^. When PicoP morphotype numbers were below our LOQ, 1.11 × 10^3^ cells mL^−1^ was used as a cell concentration and 0.89 µm (value obtained using the linear conversion above and an FSC value of zero) as median cell diameter in order to calculate morphotype and total PicoP contribution to total particulate organic carbon (POC). Cell loss due to our 24 h on ice storage approach is minimal compared to immediate fixation of a water sample. A 7–15% overestimation of PC-SYN and PEUK cells was observed in tests with water from upper estuary station NRE0. In NRE100 and NRE180 surface water, mid to lower estuary respectively, PEUK were underestimated by 7–15% at both locations, while PC-SYN were overestimated by 3% mid-estuary and underestimated by 13% in the lower estuary (Supplementary Datatable 2).

### Chlorophyll a (Chl a) measurements

Total phytoplankton biomass and PicoP biomass, phytoplankton passing through a polycarbonate < 3 µm pore-sized filter (Whatman-GE)^7^, was harvested by vacuum filtration (< 5 in. Hg) onto triplicate GF/F (Whatman-GE) filters under reduced light. The GF/F filters were wrapped in aluminum foil with biomass inward and kept in a − 20 °C freezer. Chl *a* was extracted from biomass on GF/F filters using acetone (100%) and sonication^48^. Size fractionated Chl *a* concentration was determined from a reduced set of NRE stations: 30, 50, 70, 100, 120, and 180. The samples were stored in a − 20 °C freezer between collection and analysis.

### Data plotting and analysis

Contour plots were generated using Ocean Data View 5.3.0 and using DIVA interpolation (https://odv.awi.de/)^49^. Kendall–Theil regression-nonparametric linear regression^50^ was used to evaluate the relationship between PicoP Chl *a* or % PicoP Chl *a* and Total Chl *a* via the Median-Based Linear Models (mblm) package^51^ using the R environment^52^. Values outside of 1.5 × the interquartile range plus or minus the first and third quartiles respectively were treated as outliers and removed prior to generation of linear regressions and plotting using R.

The relationship between environmental parameters and PP morphotypes abundances was studied by transformed based redundancy analysis (tb-RDA) using R^52^ and the ‘vegan’ package^53^. To reduce and identify which environmental variables (n = 12; Supplementary Datatable 1) predominantly influence observed PicoP abundances, forward selection function was implemented using the ‘adespatial’ R package^54^ with Bonferroni’s correction. The final tb-RDA plot was generated using ‘ggvegan’^55^. The relative abundance of each PicoP morphotype was transformed by Helligenger transformation prior to constructing the tb-RDA. Environmental data were standardized using Z-score transformation.

## Results and discussion

### Hydrologic and biogeochemical conditions

**Figure 2:**
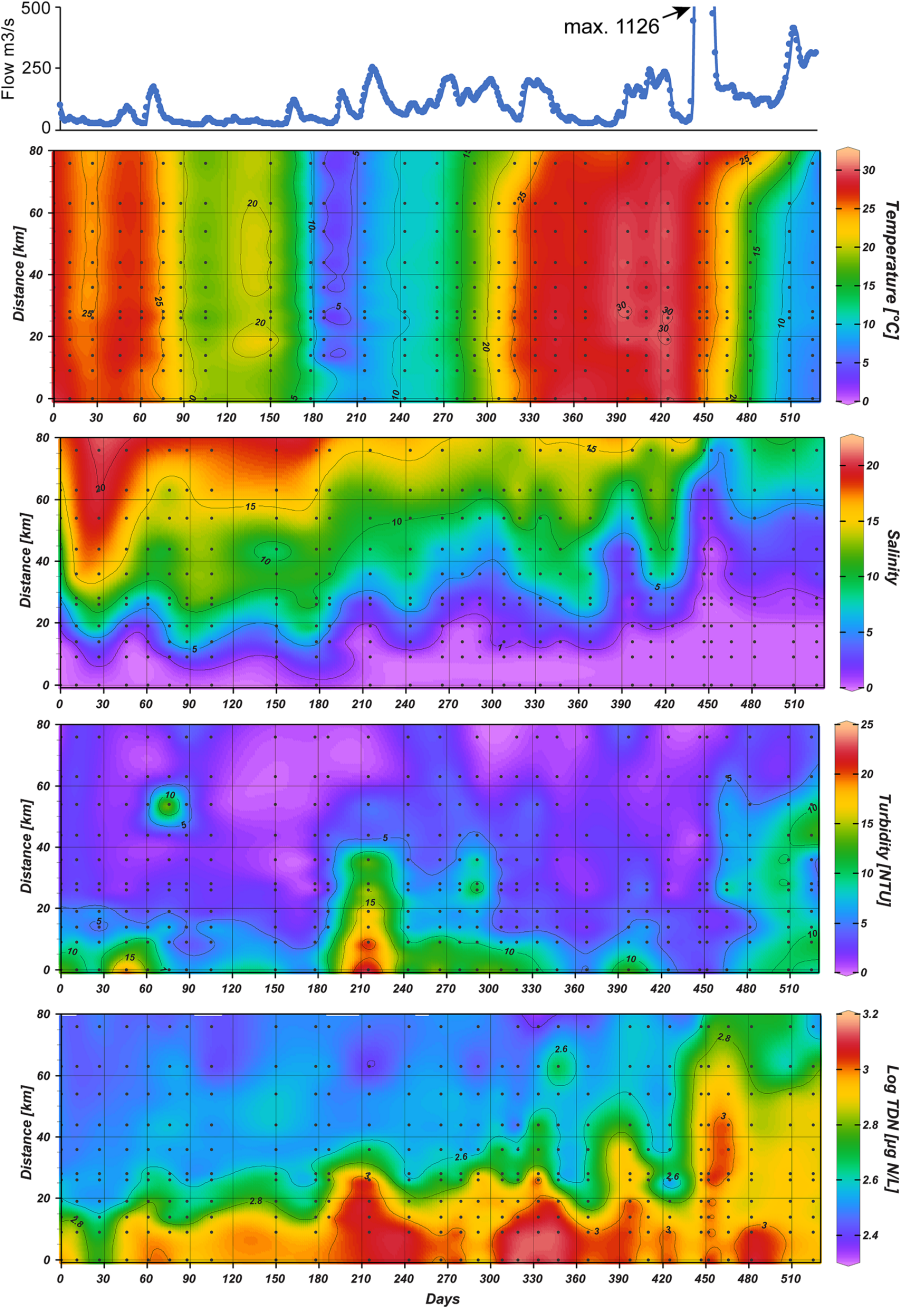
Neuse River flow rate and hydrologic conditions at NRE stations during the study period, July 2017 (study day 0) to December 2018 (study day 525). Flow data (top panel) is from the Ft. Barnwell USGS gauge, including peak flow beyond the plotted range (e.g. 1125 m^3^ s^−1^) that occurred after landfall of Hurricane Florence. Note that TDN data is log transformed.

Seasonality in hydrology and biogeochemistry, e.g. changes in temperature, primary productivity and phytoplankton biomass, was observed across NRE stations during the 525 day study (Fig. 2). For example, surface water conditions varied markedly with temperature ranging from 0.98–30.98 °C, but also salinity (0.05–21), turbidity (0.6–24.1 Nephelometric Turbidity Units (NTU)) and nutrients (e.g. total dissolved nitrogen (TDN), 239–1430 µg L^−1^) exhibited large fluctuations in the estuary, largely driven by major storm events interspersed by dry conditions (Fig. 2)^40^. Moreover, Chl *a* concentration and PP seasonally varied with highest values in spring and fall [e.g. 151 µg Chl *a* L^−1^, day 215 (February 2018)] mid-estuary (Supplementary Fig. S3), as described previously^7^.

Neuse River mean daily discharge was on average lower in 2017 than 2018, 91.3 ± 112 m^3^ s^−1^ and 178 ± 191 m^3^ s^−1^ respectively (Fig. 2; Supplementary Datatable 3). Higher river discharge in 2018 is evident based on the location of the 2.5 salinity contour which occurred > 14 km downriver the entire year (Supplementary Table S1). Overall, 2017 was a relatively dry year with mean monthly discharge for July–December being below the 1997–2019 avg. monthly means. Conversely, discharge during July–December 2018 was consistently above the 1997–2019 average monthly means except for July 2018 (Supplementary Table S1).

Landfall of Hurricane Florence on September 14, 2018 near Wrightsville Beach, NC (study day 435) caused an extreme spike in river flow reaching a maximum discharge of 1127 m^3^ s^−1^ a week later and elevated rates above 450 m^3^ s^−1^, ~ 2.5 × the annual average for 2018 two weeks after landfall (Fig. 2; Supplementary Datatable 3). Chemical conditions were also markedly altered post-Florence landfall. For example, 16 days after landfall salinity dropped from ~ 10 to ≤ 2.5 mid-estuary (60 km downstream) and remained < 7.5 three months post-Florence. High inputs of dissolved organics and nutrients were also evident^56^, e.g. colored dissolved organic matter (CDOM) concentrations increased ~ 1.75- to 2-fold relative to pre-storm levels and TDN increased ~ threefold and remained > 750 µgN L^−1^ in much of the estuary until the end of the study period (Fig. 2).

### PicoP morphotypes, abundances, and variation relative to environmental factors

**Figure 3:**
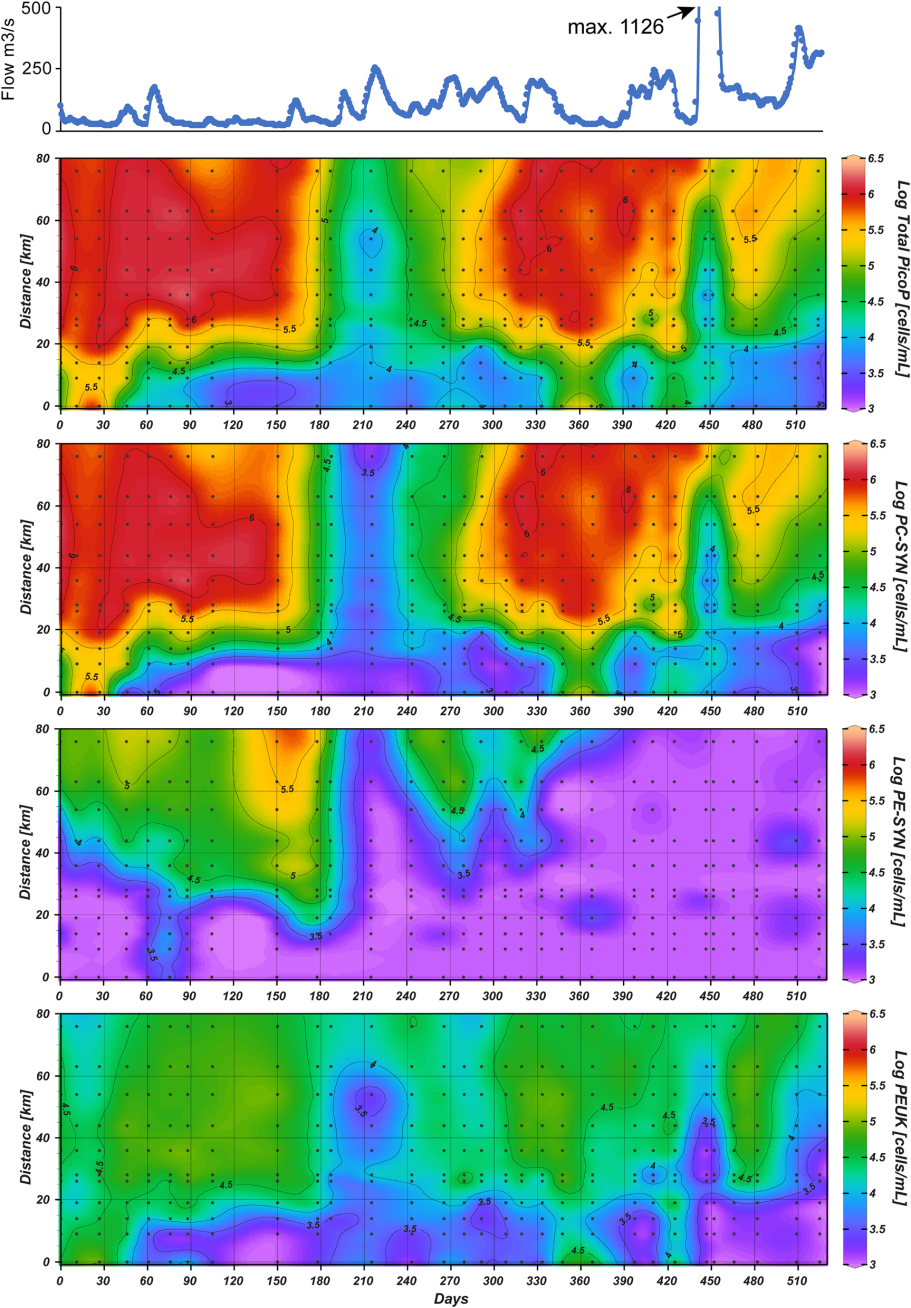
Neuse River flow rate and abundance of total PicoP and morphotypes in the NRE during the study.

PC-SYN, PE-SYN and PEUK morphotypes were prominent in NRE surface water (Fig. 3, Supplementary Fig. S1). Total PicoP abundance ranged from 2.73 × 10^3^ to 2.13 × 10^6^ cells mL^−1^ with highest abundance occurring in June to August and lowest in December to February (Fig. 3). Total PicoP abundance was often lowest in fresher regions of the estuary (e.g. < 14 km down river) with pronounced increases down river (see study days 150, 300, 400 and 475; Fig. 3).

**Figure 4:**
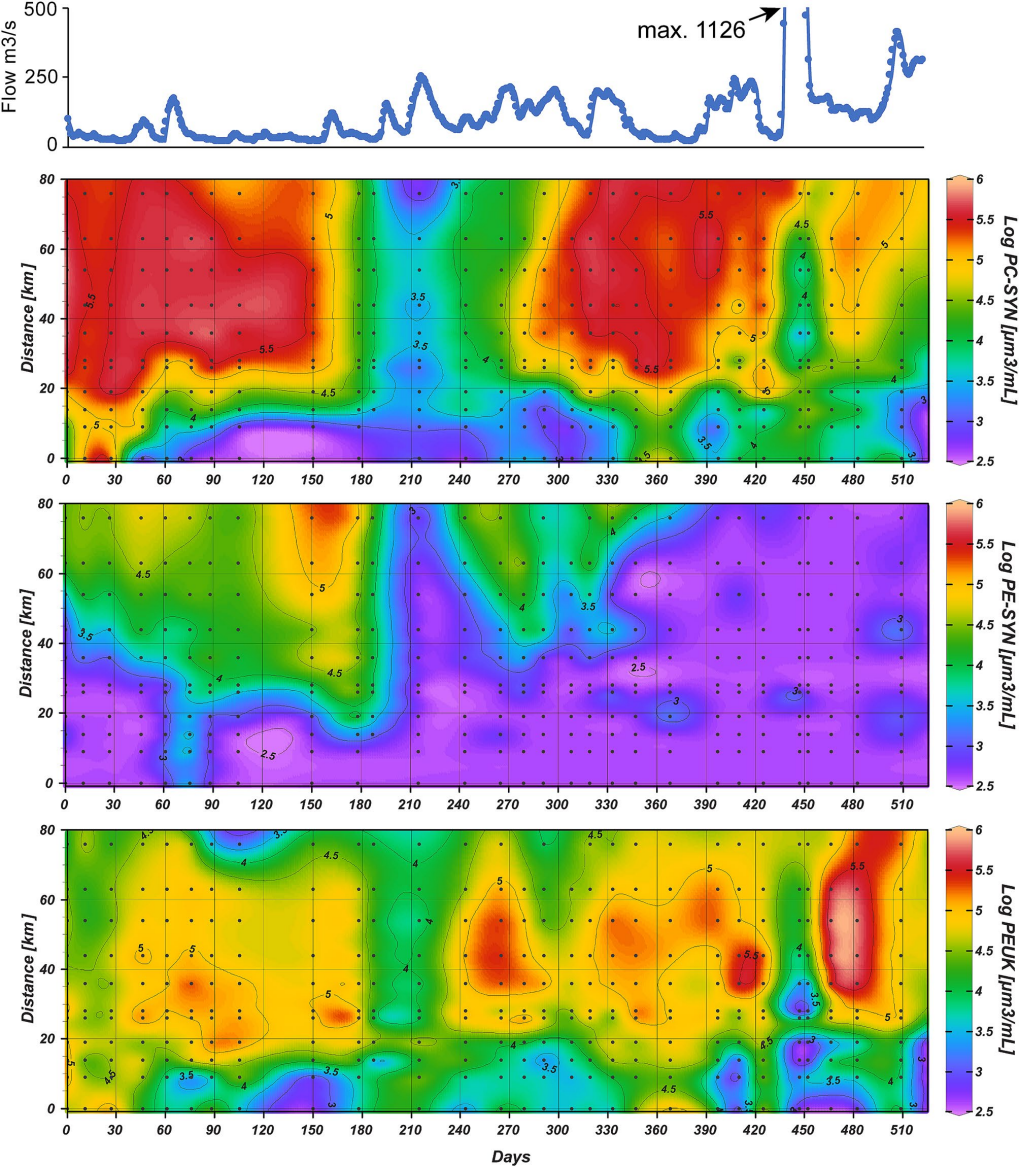
Biovolume concentrations for each NRE PicoP morphotype across NRE samplings. FCM-derived cell abundance (Fig. 3) and cell diameter (Supplementary Fig. S7) data were used to obtain biovolume per water volume. Neuse River flow rate is shown in the top panel for reference.

**Figure 5:**
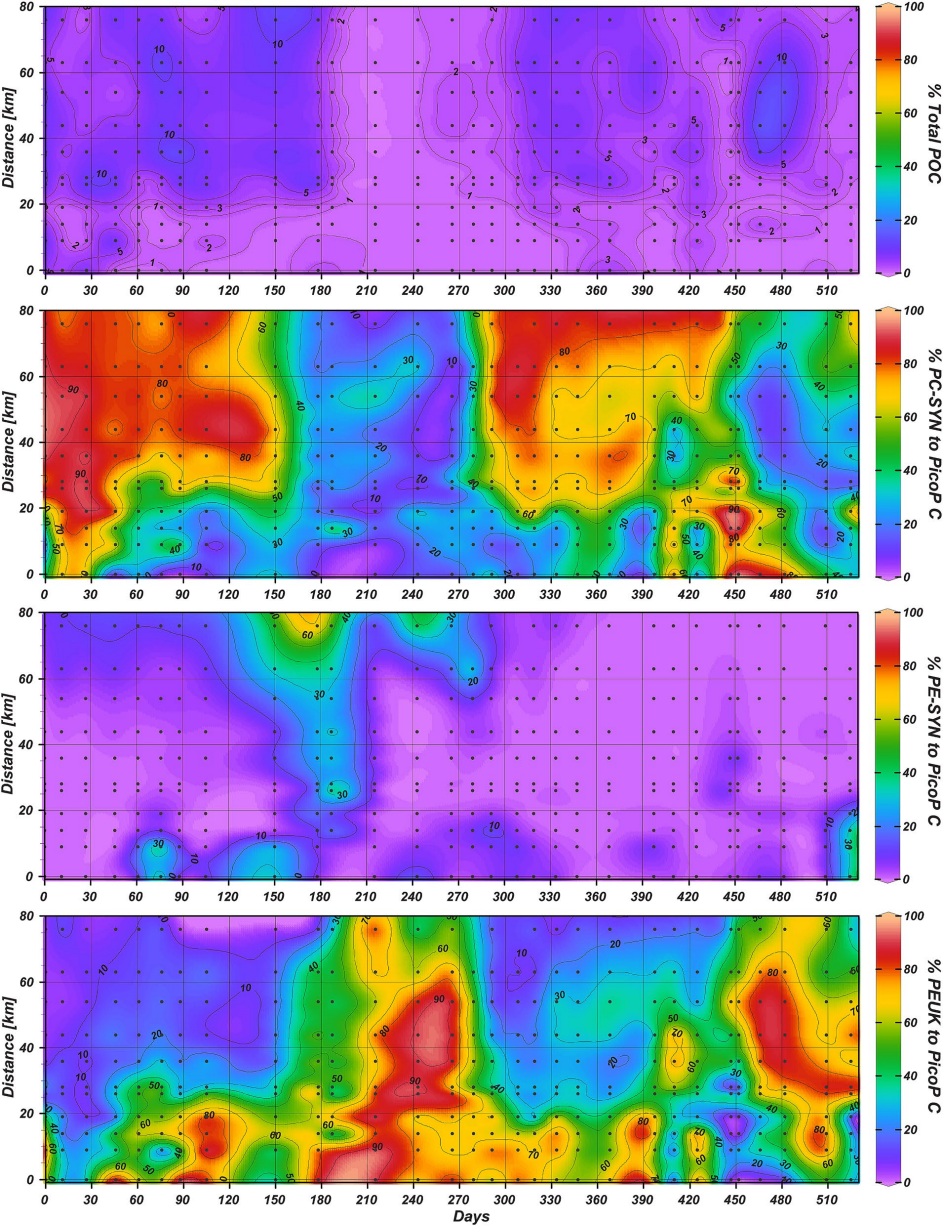
Percent contribution of PicoP to POC, as well as, percent contribution of individual PicoP morphotypes to total PicoP organic carbon.

PicoP notably accounted for 3.5% of POC on the average based on FCM-derived data and bulk POC measurements (see methods; Figs. 4, 5; Supplementary Datatable 1). The maximum PicoP contribution to POC was 16.5% and occurred mid-estuary a month after landfall of Hurricane Florence (October 15, 2018). Higher PicoP contributions to POC in general, e.g. ~ 10%, occurred in summer when total PicoP abundances were also high (Fig. 5).

**Figure 6:**
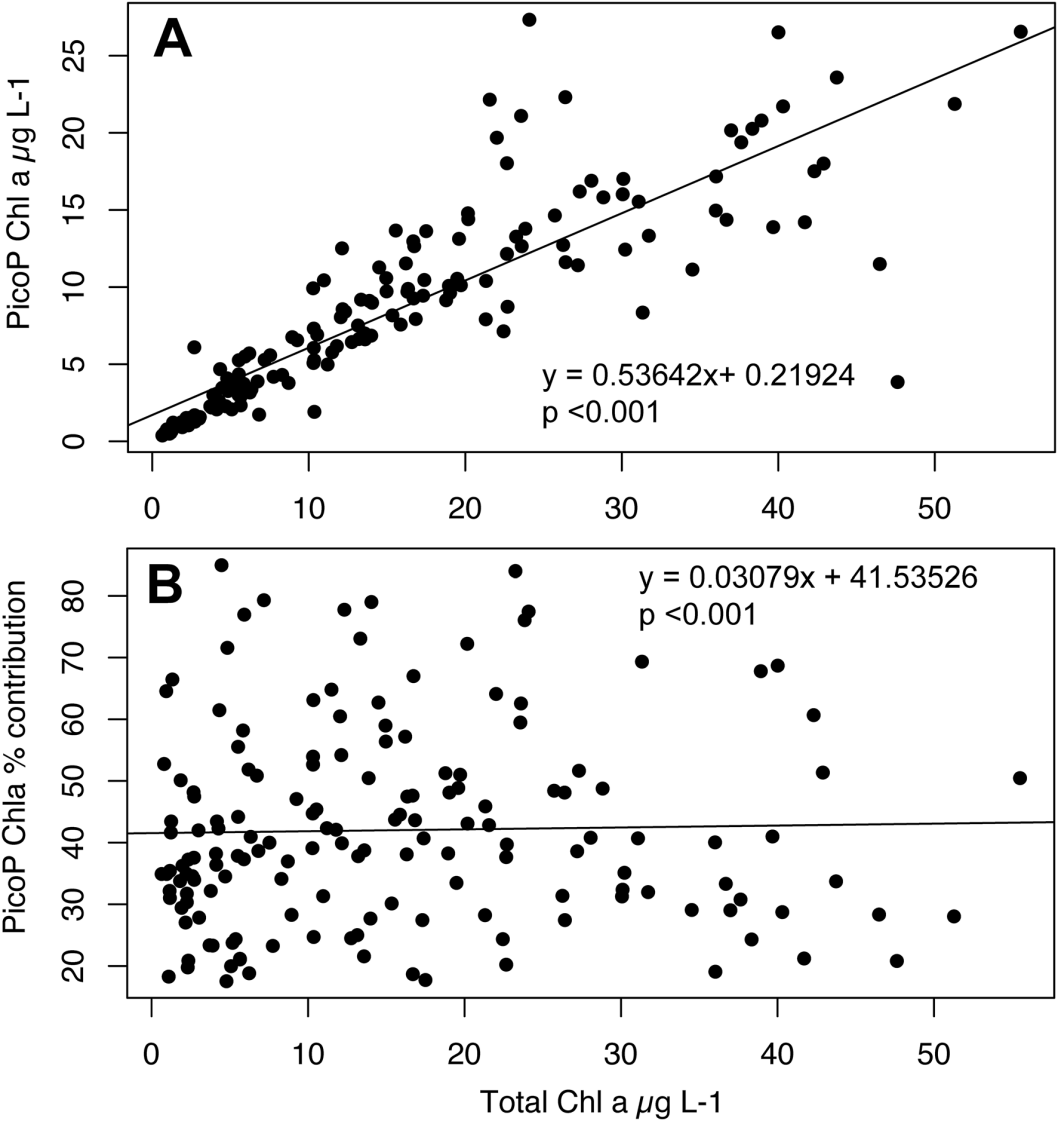
(**A**) PicoP Chl *a* concentration and (**B**) % PicoP Chl *a* concentration versus total Chl *a* concentration from six NRE stations across the freshwater to marine continuum (see “Methods”). Non-parametric linear regressions are presented and both plots were generated using R^52^.

The average contribution of PicoP to total Chl *a* concentration was ~ 45% across all samples and ranged from 0.5–100% (Supplementary Fig. S5). PicoP Chl *a* concentration generally followed change in total Chl *a* concentration, while % PicoP contribution to total Chl *a* concentration exhibited no clear relationship (Fig. 6). This suggests PicoP can account for significant amounts of total phytoplankton biomass during periods of low to high phytoplankton biomass with little competitive exclusion due to co-occurring larger phytoplankton (Fig. 6). Results from a prior NRE study show a clearer decrease in PicoP contribution with increasing total Chl *a* concentration^7^. Nonetheless, PicoP are highly significant contributors to total phytoplankton biomass (Supplementary Fig. S5)^7^ and hence key primary producers in the system.

**Figure 7:**
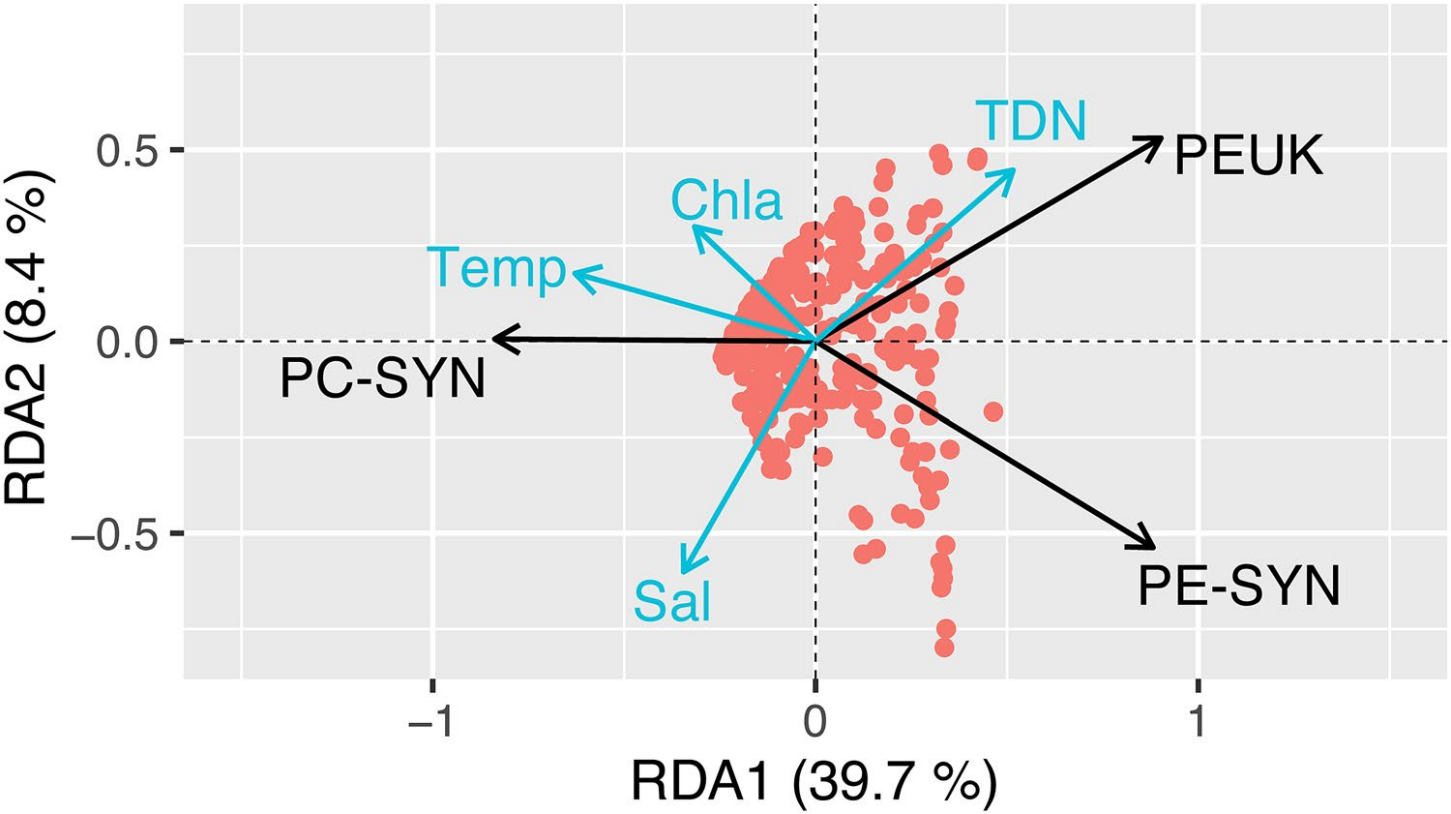
Correlation triplot from the transformed based-redundancy analysis (tb-RDA) explaining observed PicoP abundances (morphotypes; black vectors) according to environmental factors (blue vectors). Samples are represented by red circles. The triplot was interpreted as a “scaling 2—correlation biplot” where angles between variables (explanatory and/or response variables) reflect their correlations. Ordination axes were rescaled to range from − 1.5 to 1.5. All blue arrows are significant factors (Monte Carlo permutation test after a forward selection with Bonferroni’s correction, P value ≤ 0.01). Abbreviations: Temp (Temperature), Chla (Chlorophyll *a* concentration), Sal (Salinity), and TDN (Total Dissolved Nitrogen concentration). R^52^ was used to generate the triplot and color modifications were made in Adobe Illustrator 23.0.1.

Based on tb-RDA multivariate analysis 45.6% of PicoP morphotype abundance variance can be explained by significant environmental variables (Supplementary Table S2), suggesting factors not measured in this study likely have a significant impact on PicoP morphotype abundances. Of all the measured environmental parameters, TDN concentration (Monte Carlo test-Pseudo F = 86.137; *P* = 0.012) and temperature (Monte Carlo test-Pseudo F = 105.314; *P* = 0.012) had significant impacts and explained 16% and 24% of variance in PicoP morphotype abundances respectively. Briefly summarizing the tb-RDA analysis, PC-SYN abundance was positively related to temperature and Chl *a* concentration while PE-SYN abundance was negatively related to these two environmental variables. PEUK abundance was positively related to TDN concentration and negatively related to salinity and temperature (Fig. 7).

### PC-SYN dominate PicoP cell abundance and biomass

PC-SYN were the most abundant NRE PicoP across the study and reached a maximum of ~ 10^6^ cells mL^−1^ in summer. Overall, PC-SYN were also the largest contributors to PicoP biomass (Figs. 3, 4, 5) and often outnumbered PE-SYN by more than two to three orders of magnitude (Supplementary Fig. S6). In other high residence time (≥ 1 mo.) estuaries and coastal bays the PC-SYN:PE-SYN ratio is around 10 in mesohaline to polyhaline waters^16,57,58^. The observed dominance of PC-SYN emphasizes their importance in large temperate estuarine systems. Notably, PC-SYN have received less recent attention than PE-SYN in estuaries and coastal seas^8,12,17,57,59^.

The size of NRE PC-SYN is smaller (median diameter = 0.94 ± 0.19 µm) (Supplementary Fig. S4) than bloom forming *Synechococcus* reported from Florida Bay (~ 2 µm diameter) as well as Chesapeake Bay *Synechococcus* (mean = 1.5 µm)^8,60^. Considering bacterial cell size positively correlates with genome size^61^, NRE PC-SYN may be genetically distinct. Diverse lineages of PC-rich Picocyanos, including *Synechococcus, Synechocystis and Cyanobium*, occur in aquatic systems and several lack representative genomes or isolates in culture^17,29,59,62,63^. Accordingly, the ecophysiology and metabolic capacities of PC-SYN lineages are not well resolved beyond pigmentation^59,63,64^ and possession of toxin-antitoxin systems^65^. Filling this knowledge gap will potentially help explain the success of PC-SYN in estuaries but also their observed detrimental effects on co-occurring filter-feeders (e.g. sponges), and ability to avoid predation and viral lysis, which alters energy and nutrient flow in the microbial food web^37,60,66^.

Meso- and polyhaline waters (salinity ~ 5–20) were rich in NRE PC-SYN, while fresher waters < 1 salinity contained notably lower numbers (Fig. 3; Supplementary Fig. S5). More spatially coarse data from tropical and temperate estuaries, Pensacola Bay and Pearl River Estuary, suggest a similar trend in PC-SYN abundance^16,17^. This pattern is in agreement with observed reductions in zeaxanthin during increased river discharge and a prior suggestion that NRE cyanobacteria are more susceptible to flushing compared to other phytoplankton^32,67^. The physiochemical reasons for this higher susceptibility to flushing remain unclear. A lack of key ions (e.g. Na^+^, Mg^2+^, Cl^−^) could constrain PC-SYN habitat; however, estuarine isolates can grow in freshwater medium^68^ and PC-rich cells are prevalent in diverse freshwater systems^3,69,70^. Several factors that covary with salinity could singularly or collectively limit PC-SYN, these include increased CDOM concentrations^71^, which alters light quality and intensity^64,72^, composition or activity of co-occurring bacterivorous grazers^34,35,73^ and increased heavy metal concentration^74^. Generally, PC-SYN are well-adapted to red-shifted and low light conditions^57^, but light limitation of NRE PicoP has not been investigated. With regard to grazing, nano- to micro-zooplankton can exert high grazing pressure on NRE PicoP^33^. Laboratory-based experiments show that select grazers preferentially consume certain PicoP^34,35^. Potentially grazers in fresher regions of the NRE exert higher grazing pressure on PC-SYN cells and keep their abundance low. Concentrations of cyanotoxic heavy metals, esp. copper and cadmium^74^, tend to increase with decreasing salinity in estuaries^75^. Elevated heavy metal concentrations are blamed for reduced Picocyano numbers in coastal Mediterranean^76,77^. Exceedances of copper (> 3 µg L^−1^) occur in the upper to mid NRE (e.g. NRE30, NRE120, NRE140; Fig. 1)^78^, but physiological experiments testing PicoP copper toxicity and more frequent measurements of surface water copper concentrations (and other metals) are needed to confirm this hypothesis.

PE-SYN were prevalent, e.g. reaching a maximum 4.1 × 10^5^ cells mL^−1^, in higher salinity waters—an expected result based on prior estuarine studies^8,12,16,17^. Generally, PE-SYN were outnumbered by PC-SYN and contributed a minimal 4.8% on the average to PicoP organic carbon (Fig. 5; Supplementary Datatable 1). During winter to spring at saline downriver stations the PE-SYN contribution to PicoP organic carbon spiked and reached up to 68% (see study days ~ 150–190, 240; Fig. 5). The NRE PE-SYN detected are likely PE-rich *Synechococcus* which are common in coastal ocean waters, e.g. Marine Subgroup 5.1 affiliates^17,29,79,80^ or recently described Marine Subgroup 5.2 affiliates with PE-encoding genes^59^.

### PEUK: unexpected and significant contributors to estuarine PicoP biomass

In contrast with PE-SYN, PEUK exhibited more spatial and temporal overlap with PC-SYN (Fig. 2). PEUK also inhabited fresher NRE waters, which emphasizes their ability to thrive in oligohaline to mesohaline waters (Fig. 2). PEUK were more resilient to temperature fluctuation than PC-SYN as their abundance decreased less from fall into winter than other PicoP at downriver stations (see study days 180–215; Fig. 2). The results of our multivariate analysis also reflect this with distinct positioning of PEUK versus other PicoP vectors relative to temperature and salinity (Fig. 7).

PEUK were larger, 1.54 ± 0.51 µm median diameter, than other PicoP, which is congruent with data from other estuarine and marine systems (Supplementary Figs. S4, S7)^11^. However, large and small PEUK were evident (Fig. 4; Supplementary Fig. S7). Cells ~ 1–1.25 µm in diameter were prevalent in mesohaline waters during summer to winter, while cells > 2 µm were dominant in oligohaline waters during spring ‘blooms’ and after periods of high river flow (Fig. 4; Supplementary Fig. S7). Similarly, in Mediterranean coastal systems larger (> 2 µm diameter) PEUK bloom in eutrophic and hypereutrophic lagoons, whereas smaller (< 1 µm diameter) PEUK dominate in oligotrophic to mesotrophic lagoons^27^. FCM is insufficient to distinguish PicoP genera^11^. Nonetheless, the small PEUK cells are likely *Ostreococcus*, *Micromonas*, or other small Chlorophytes that are ~ 1 µm in diameter and common in estuaries and coastal ocean waters^3,24,44^. Larger PEUK cells are presumably different Chlorophytes or Haptophytes. Diagnostic photopigment data suggests Chlorophytes and Cryptophytes are common in the NRE and accordingly increase in numbers in response to elevated river flow and nutrient inputs^32,67^. Metatranscriptomic libraries from the NRE contain Chlorophyte and Haptophyte sequences belonging to *Emiliania*, *Phaeocystis*, *Ostreococcus* and *Micromonas* spp.^81^, all of which are candidate PEUK populations detected here by FCM.

While PEUK were less abundant than PC-SYN, they reached a maximum of 9.8 × 10^4^ cells mL^−1^, which at times accounted for upwards of 90% of PicoP organic carbon (Figs. 2, 5). These high PEUK contribution periods were during winter to spring, in mid-estuary in association with increased river discharge events during summer/fall before the seasonal drop in temperature below 10 °C in late fall and in oligohaline regions across multiple seasons (Figs. 2, 5). PEUK, Picocyanos and total PicoP abundance and biomass all declined with the seasonal temperature drop and high discharge storm events in summer and fall. However, abundance and biomass of PEUK, and PC-SYN to a degree, recovered following the storms. In this recovery period, PicoP contributed more to total POC, ~ 5–10%, with PEUK accounting for more of PicoP biomass (see study days 425, 466 and 482; Figs. 2, 3, 5). In contrast, PEUK were significant contributors to PicoP biomass during the spring bloom, but the PicoP contribution to total POC was lower, e.g. a maximum of ~ 2%, due to co-occurring larger phytoplankton (see study days 215, 265; Fig. 5, Supplementary Fig. S3).

The occurrence of PEUK in the NRE is not surprising since they are common in diverse estuaries and coastal lagoons^3,12,13,19,44^. Yet, PEUK have garnered markedly less attention than Picocyanos in large temperate US estuaries^7,8,16,82^. The data presented here highlight PEUK as significant primary producers that require consideration as contributors to energy and nutrient flow up the estuarine food web^33^. PEUK are generally recognized as important marine phytoplankton due to their significant contributions to total phytoplankton biomass and productivity in oligotrophic and pelagic waters^24,83–85^, their desirability as prey^22,73,86^ and strain specific bloom events in coastal waters that negatively impact ecosystem health^87,88^. Top down and bottom up controls dictate PEUK biomass and community composition—e.g. nano- and microzooplankton grazing rates, light intensity and quality, mixotrophy, use of inorganic and organic nutrients, trace metal concentrations, and temperature^86,89–95^. PEUK tend to be more prevalent under mesotrophic to eutrophic conditions^19,27^, elevated mixing/turbidity^19,26^, as well as warmer temperatures^24,44^—although marine PEUK can thrive under cooler conditions relative to Picocyanos and peak in abundance earlier in the season^19,92^. In agreement with these environmental trends, PEUK exhibit higher maximum growth rates relative to Picocyanos and nanophytoplankton^44^ and prefer reduced nitrogen^87^. Our observations of higher PicoP biomass contributions by PEUK during cooler periods and periods of increased river discharge fits with prevailing views of PEUK ecology, yet further studies are needed to definitively resolve the key bottom-up and top-down controls and how they relate to increased river discharge, e.g. disruption of grazing through mixing^26^ or increased heavy metal loads^76^.

### Moderate and extreme precipitation reduces PicoP biomass and alters composition

Moderate precipitation storm events, which are operationally defined here by river flow exceeding the annual average of 178 m^3^ s^−1^, negatively impacted total PicoP numbers and biomass in 2018. PicoP abundance rarely exceeded 10^6^ cells mL^−1^ in 2018 and abundances > 5.5 × 10^5^ cells mL^−1^ occurred more down river (Fig. 3). Excluding data from after landfall of extreme storm Hurricane Florence, PicoP biomass was ~ twofold higher during drier 2017 versus wetter 2018, e.g. 8.2 × 10^3^ µgC L^−1^ vs. 4.5 × 10^3^ µgC L^−1^ (Supplementary Datatable 1; Fig. 5).

High precipitation-containing Hurricane Florence dramatically reduced PicoP abundance and biomass by ~ 100-fold weeks after landfall (see study day 450; Fig. 3). Discharge remained > 500 m^3^ s^−1^, ~ 3 × higher than the average for 2018, during this period and peaked at 1126 m^3^ s^−1^ (Fig. 1; Supplementary Datatable 3). This elevated discharge also resulted in notable declines in Chl *a* concentration and PP (Supplementary Fig. S4). One month after Florence PC-SYN and PEUK cell abundances rebounded in the lower to mid-estuary alongside Chl *a* concentration and PP (Fig. 3, Supplementary Fig. 4). The recovery highlights PicoP and large phytoplankton resilience despite 16 days of elevated river discharge above the 2018 average of 178 m^3^ s^−1^ and minimal intrusion of saltier Pamlico Sound water (Figs. 1, 2). However, PicoP numbers post-Florence leveled off at abundances 2- to 10-fold lower than those seen a year prior in the mid to lower estuary (see study day 150 vs. 480, Fig. 3), suggesting the extreme precipitation event had an extended impact on PicoP for at least a month.

Increased precipitation in the NRE watershed also dramatically altered PicoP community composition. PE-SYN were three orders of magnitude less abundant than PC-SYN in the lower estuary in the wetter 2018, as well as lower PC-SYN abundance, and increased abundance in larger PEUK that appeared as ephemeral “blooms” (Figs. 3, 4). Strikingly, PEUK cell abundance and size increased post-Florence in mid-estuary (Fig. 3). Periods of relatively high rainfall prior to Florence around study day 400 led to increased river discharge ~ 250 m^3^ s^−1^ and ultimately increases in PEUK abundance, size and contribution to PicoP biomass in the upper to mid-estuary (Figs. 4, 5). A link between increased riverine inputs promoting larger PEUK populations relative to Picocyanos agrees with previously reported negative correlations between salinity and Picocyano abundance in tropical and temperate estuaries^13,16,17^, but also declines in cyanobacteria and increases in eukaryotic phytoplankton in the NRE following increases in river discharge^32,42,96,97^. Shifts in PicoP community composition can potentially impact ecosystem health and function in addition to modifying the estuarine carbon cycle. Specifically, PicoP community composition potentially: (1) alters energy and nutrient flow in the food web as PicoP vary in their susceptibility to grazing^73,86,98^ and viral infection and lysis^99–101^, (2) changes retention of nutrients entering the estuary as PicoP vary in their abilities to assimilate nutrients, including inorganic and organic N, P^102–105^ and (3) alters behavior or health of co-occurring organisms as select PicoP produce secondary metabolites and allelopathic compounds^36,38,106,107^.

TDN concentration, a variable closely linked to riverine discharge^32^, explained 15.9% of PicoP abundance variance based on multivariate analysis (Fig. 7; Supplementary Table S2). Only temperature explained more variation at 24.5%, which is not surprising given the well-documented seasonal change in estuarine and marine PicoP abundance, esp. Picocyano abundance^7,16,80,108^. TDN itself is not thought to be a critical modulator of PicoP abundance. The relationship with TDN is interpreted to reflect the impact of other covariables associated with river discharge, e.g. increased dissolved organics altering the light field^72^, greater mixing or higher concentrations of inhibitory compounds not measured here (e.g. heavy metals). Turbidity, inorganic and organic N and P concentrations and POC also increase with river discharge to a degree^7,32^, but were minimally explanatory based on our analysis (e.g. R2 < 1%; p values > 0.05; Supplementary Table S2). These results point to increased precipitation as a key modulator of PicoP abundances and community composition on week to month as well as annual time scales (Figs. 2, 3, 7).

A higher frequency of extreme precipitation events (tropical cyclones) and more precipitation associated with non-cyclone storm events between extensive droughts is predicted in the regions surrounding the NRE, but also other globally distributed systems—making precipitation frequency a regional to global concern^109–112^. This is particularly true for estuaries and coastal systems where plankton community structure and biomass are altered by storms of varying severity^113–115^. Based the data presented here and prior results from other estuaries^13,16,17^, reductions in total PicoP biomass and promotion of larger PEUK are expected in temperate/tropical estuaries poised to receive more precipitation via moderate to extreme storms.

## Conclusion

Despite being an understudied group of estuarine and marine PicoP, PC-SYN are key primary producers in a major tributary of the second largest US estuary, which emphasizes their general importance in large, long residence time estuaries. PEUK deserve greater attention in the NRE and possibly other large temperate estuaries. PEUK contributions to biomass in winter to spring and after periods of moderate to extreme riverine inputs is of particular ecological importance as these are times when increased productivity is followed by increased grazing and upward support of the food web.

Speculatively, PicoP are often overlooked phytoplankton in estuaries because their largest contribution to PB and PP is generally in oligotrophic and pelagic systems^3,116,117^. There is large variability though in PicoP PB and PP contribution data from coastal systems (e.g. 1–90%) as well as the size fractionation methodology (e.g. filter pore-size cut-offs) used to estimate these contributions^3^. The lack of attention to estuarine PEUK is particularly intriguing and may be due to difficulty distinguishing them from PC-SYN via microscopy^118^, limited temporal/spatial sampling or greater focus on PicoP abundance rather than biomass^116^.

While the contribution of PicoP to PP and PB in estuaries is becoming clearer based on the data presented here and other recent findings^5–8,13^, knowledge of their biogeochemical and ecological roles is arguably lagging behind. Currently more is known about their roles in pelagic marine waters^89,90,102–104^. Experiments with environmentally relevant isolates and natural populations would provide insight on the responsiveness, resilience and ecophysiology of estuarine PicoP, especially PEUK populations. This is particularly important to investigate in the context of a changing climatic and hydrologic “state change”^40^ (esp. pre- and post-storm events) and will be crucial for prediction and quantification of their contributions to the food web^33^ and microbial loop (esp. labile DOM)^119^.

Consideration of climate-change associated impacts on marine PicoP has primarily focused on the potential consequences of rising temperature on PicoP in non-brackish coastal and open ocean waters^120–122^. Our results highlight precipitation as an equally important source of change to PicoP within the NRE, and perhaps more broadly, in large high-residence time estuaries. The results follow the paradigm that higher flushing times reduce total phytoplankton biomass in estuaries^123^, while highlighting the consequences of a ‘wetter future’ where more frequent moderate to extreme precipitation events will alter ecosystems, even down to the smallest of phytoplankton.

## Supplementary Information

Supplementary Information 1.

Supplementary Information 2.

Supplementary Information 3.

Supplementary Information 4.

Supplementary Information 5.
