# Supplementary material for: Picophytoplankton dynamics in a large temperate estuary and impacts of extreme storm events
Source: Sci Rep. 2020 Dec 16;10:22026. doi: 10.1038/s41598-020-79157-6 (PMC7744581; doi:10.1038/s41598-020-79157-6)
Supplement: Supplementary file 1 — Supplementary Information 1. [file 41598_2020_79157_MOESM1_ESM.pdf]

Supplementary material for:

Picophytoplankton dynamics in a large temperate estuary and impacts of extreme storm events

Ryan W. Paerl[1], Rebecca E. Venezia[1], Joel J. Sanchez[1], Hans W. Paerl[2]

[1]Department of Marine Earth and Atmospheric Sciences, North Carolina State University, Raleigh, North Carolina 27695-8208, United States of America

[2]Institute of Marine Sciences, University of North Carolina at Chapel Hill, Morehead City, NC 28557, United States of America

Figure S1. Example flow cytograms and PicoP gating used in analysis of NRE samples. In (A) PE-SYN cells are distinguished based on yellow fluorescence from blue excitation light (Yellow-B) vs. red fluorescence from blue excitation light (Red-B). In (B) PC-SYN and PEUK cells are distinguished based on red fluorescence from red excitation light (Red-R) versus red fluorescence from blue excitation light (Red-B). PC-SYN cells exhibit notably higher Red-R signal relative to Red-B. (C) A secondary gate of PE-SYN events was applied for all samples based on forward- and side-scatter signals, to exclude larger (up and right) events occurring within the SYN-PE gates that also occurred in deionized water blanks (D).

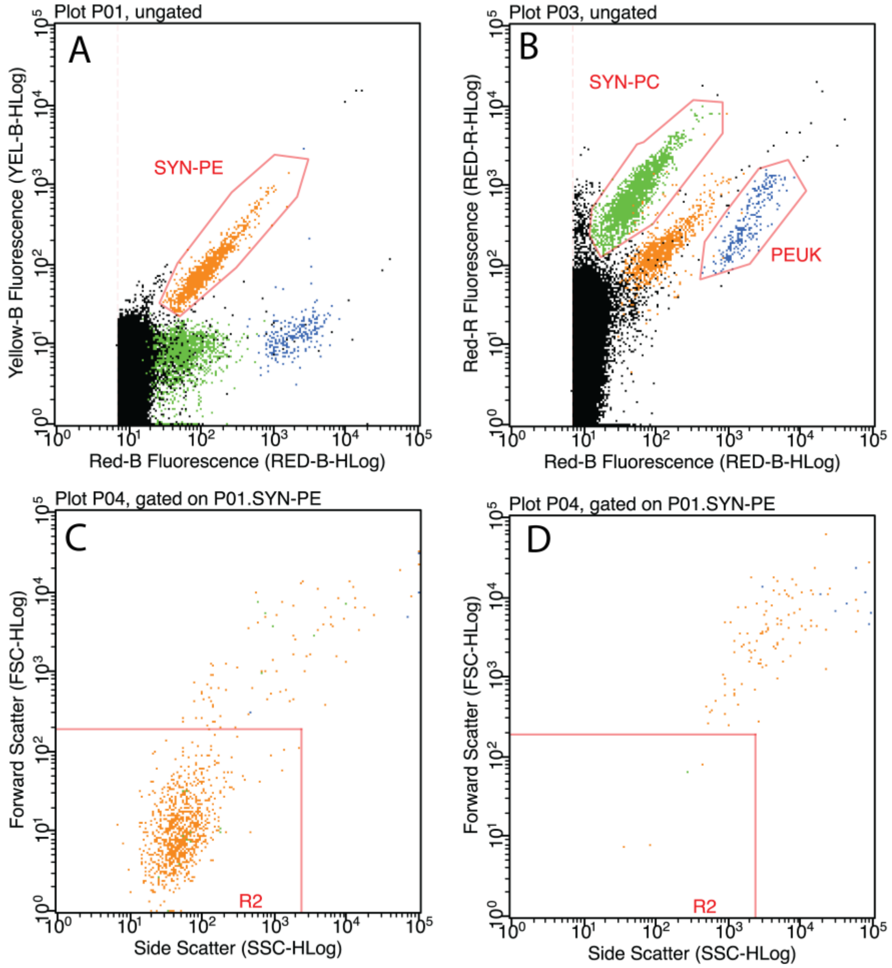

Figure S2. Mean forward scatter (FSC) of reference beads with mean diameters of 0.5, 2, 3.3, and 5.11 µm (n ≥ 75 for each reference bead). A linear calibration curve was used to determine the cell diameter of PicoP based on their measured forward scatter values (FSC).

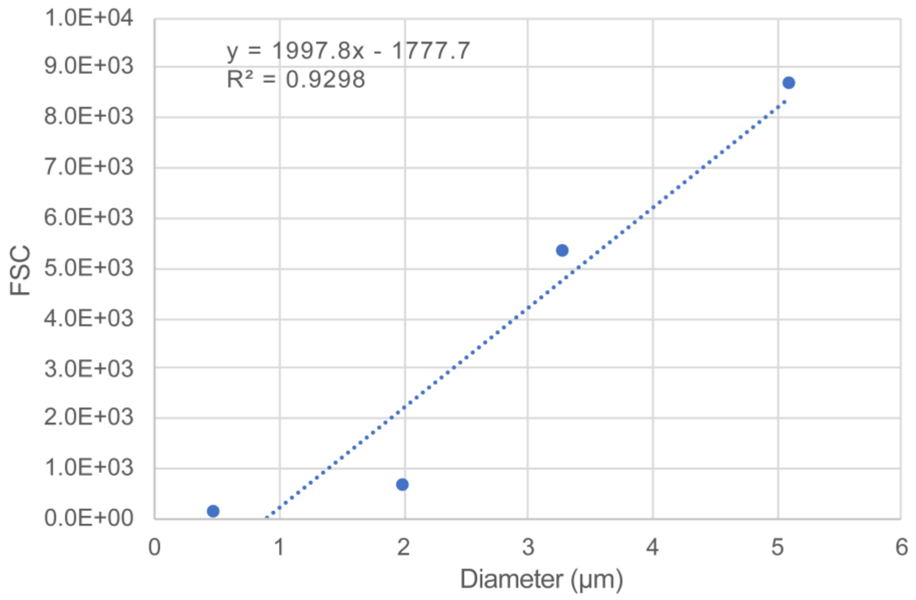

Figure S3. Neuse River flow rate (Ft. Barnwell Station), Chlorophyll *a* concentration, and primary production rate from all MODMON stations during the 2017-2018 study.

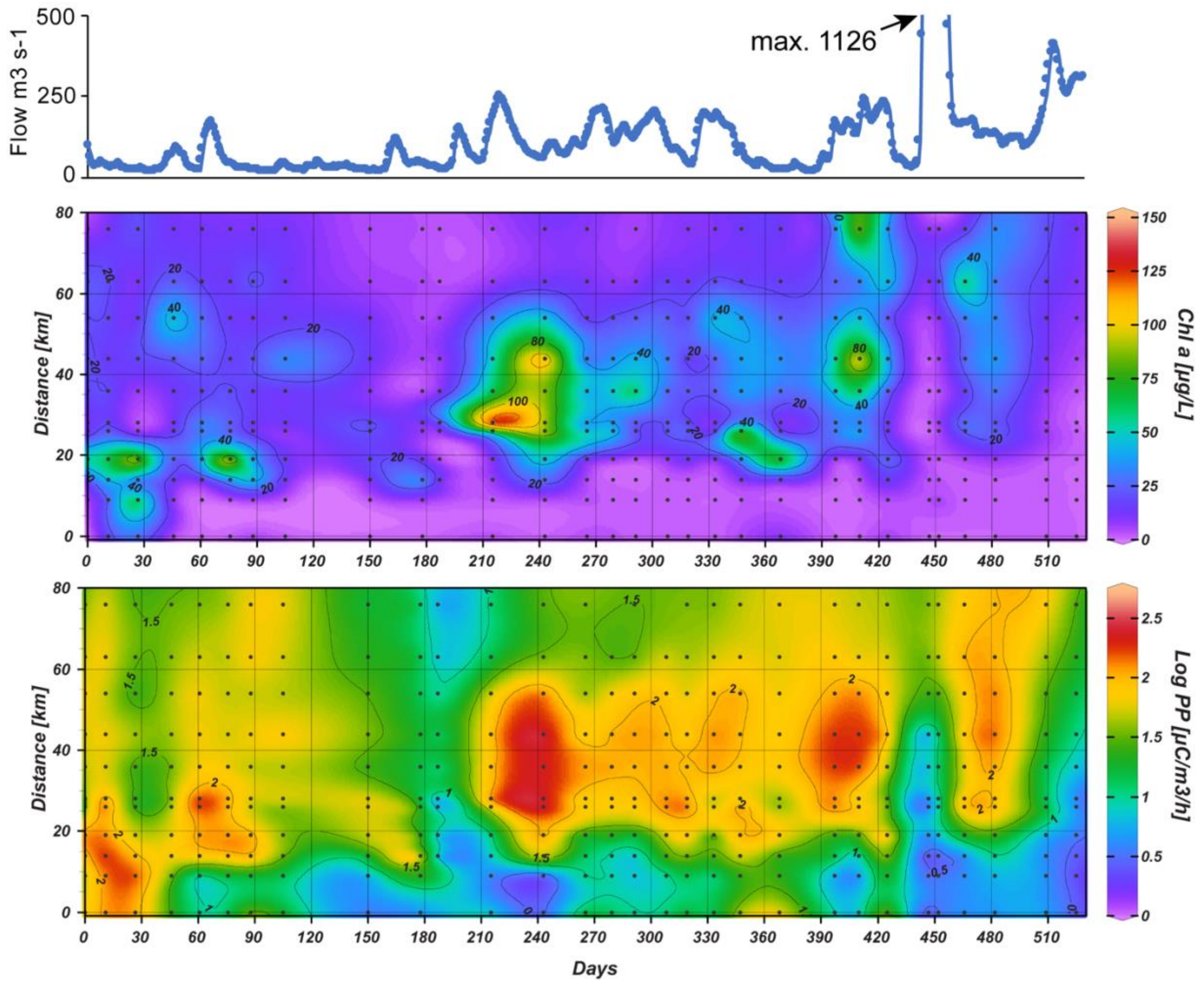

Figure S4. Box plots representing the median cell diameter (μm) for each morphotype present in the NRE across all samples and respective standard deviations. For PE-SYN *n* = 121; for PC-SYN *n* = 302; for PEUK *n* = 293 and vary given that some morphotypes were below the limit of quantification in some samples.

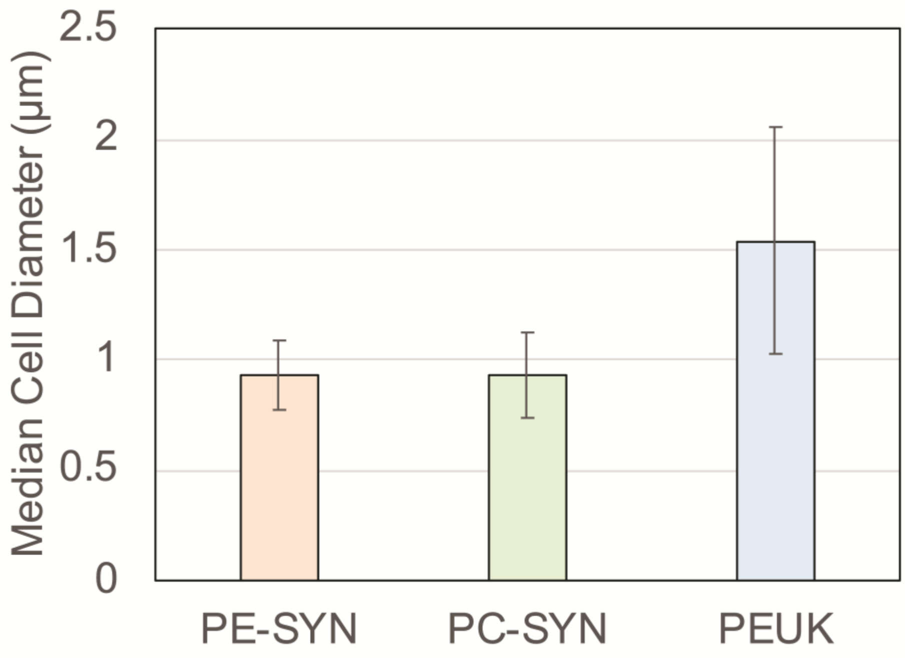

Figure S5. Neuse River flow rate (Ft. Barnwell Station), PicoP Chlorophyll *a* concentration based on size-fractionated filtrations (see methods) and percent contribution by PicoP to total Chlorophyll *a* concentration. Measurements of size fractionated Chlorophyll *a* concentration were collected from select MODMON stations leading to coarser contour resolution relative to Figure S3 (MODMON collected Chlorophyll *a* concentration data).

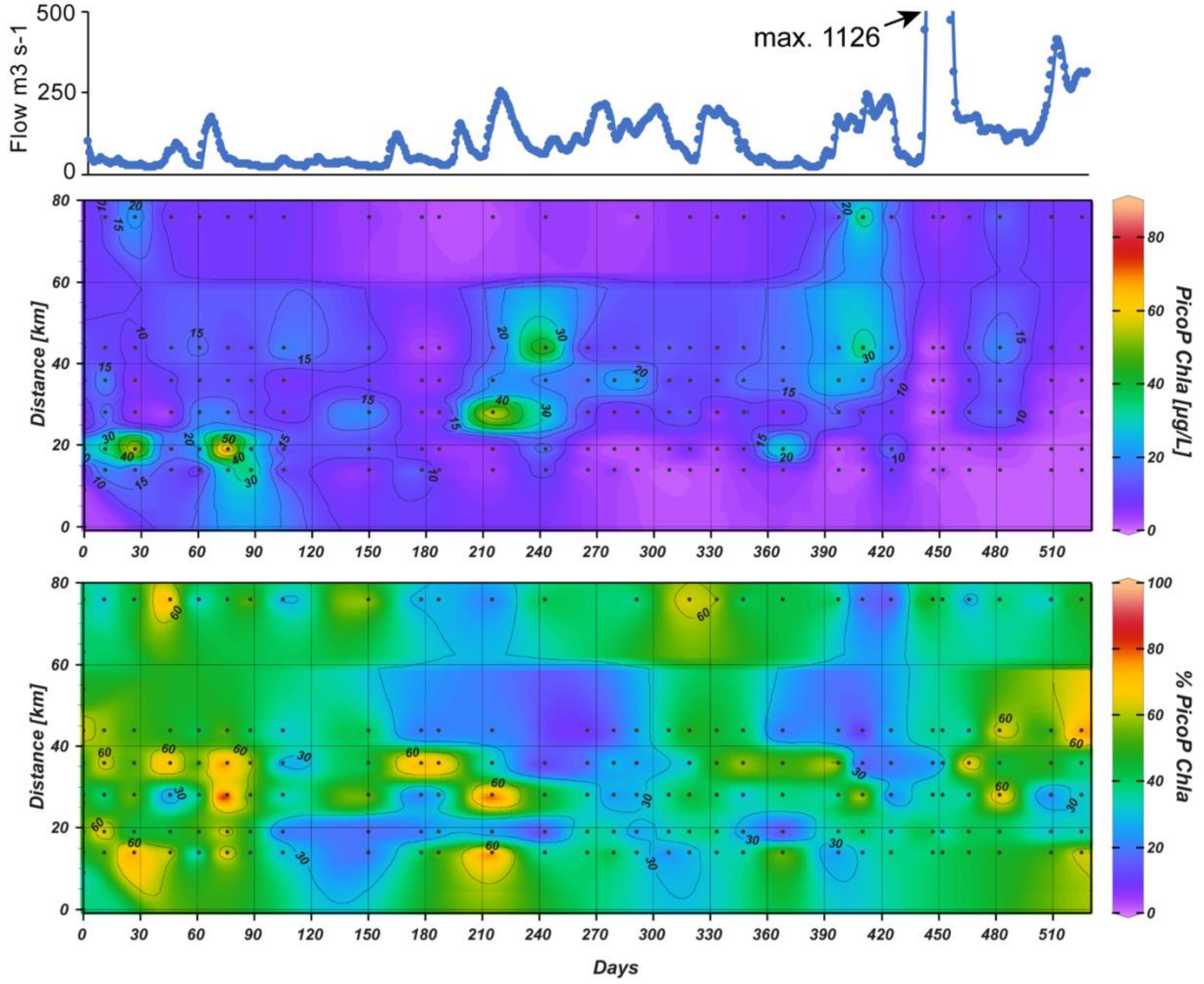

Figure S6. Change in the ratio of PC-SYN:PE-SYN with respect to salinity in the NRE. A power fit of FCM data from salinity ≥5 (circles) is provided, similar to the approach drawn by eye in Murrell and Lores (2004). Notably, the ratio slightly declines with increasing salinity, yet the power fit poorly represents the data (R^2 = 0.1013; PC-SYN:PE-SYN data are non-normal in their distribution based on a Shapiro-Wilk normality test). The dashed grey line represents a 1:1 ratio for reference.

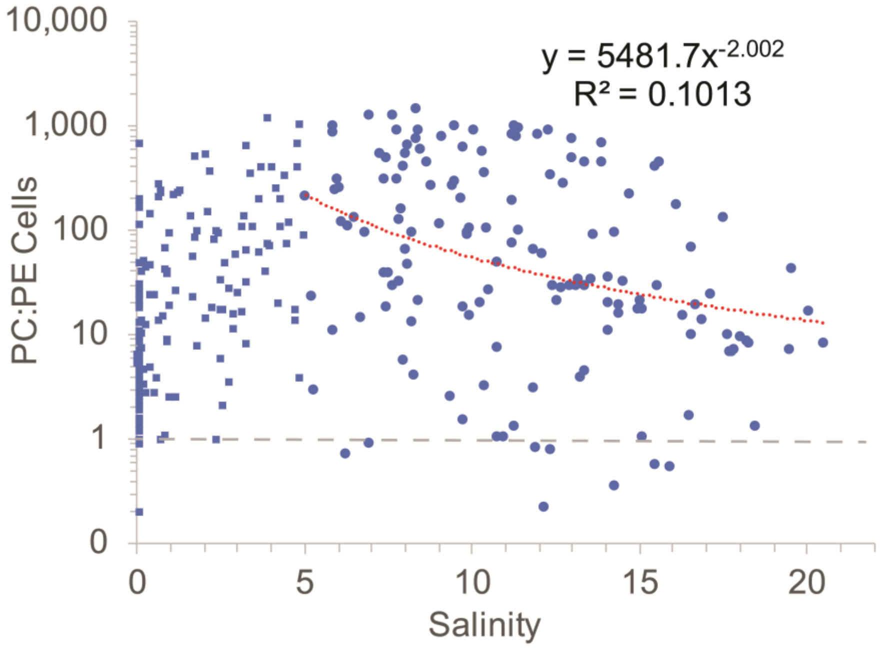

Figure S7. Cell diameter of PicoP morphotypes during the study with Neuse River flow rate for reference.

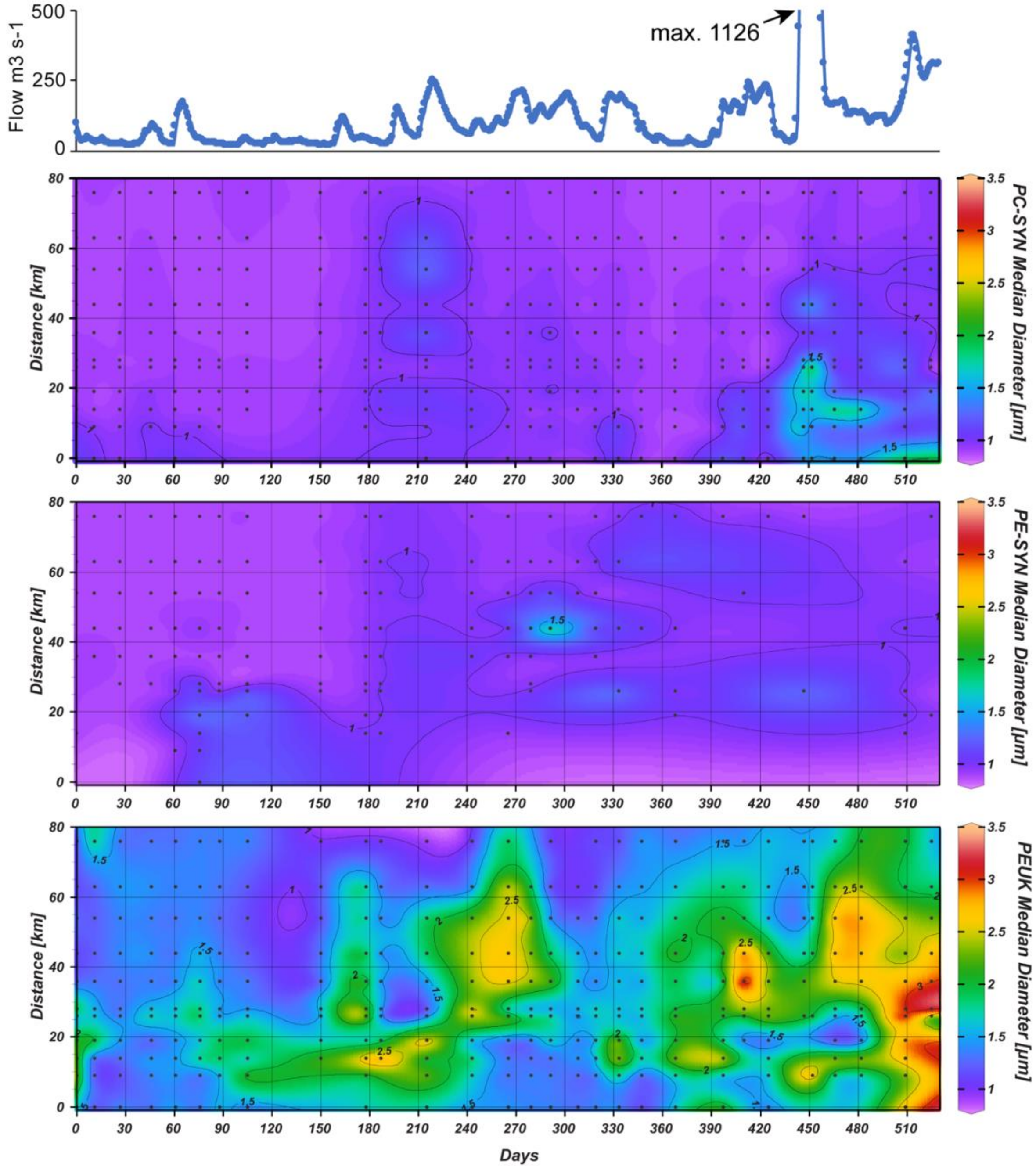

Table S1. Mean monthly discharge rate ($m^3\ s^{-1}$) of the Neuse River at Ft. Barnwell (USGS Station 02091814) during 1997-2019, as well as 2017 and 2018 individually. Shaded rows emphasize data from single years.

	**Jul**	**Aug**	**Sep**	**Oct**	**Nov**	**Dec**
2017	49	42	69	26	30	52
2018	38	181	501	147	211	365
1997-2019 mean	67	71	125	114	86	119
1997-2019 stdev	72	58	170	164	76	85

Table S2. Variance terms and respective significance values associated with tb-RDA analysis of PicoP abundances with respect to measured environmental variables. Variables with adjusted p-values (pval.adj) < 0.05 are shown and were used for RDA model testing. Twelve environmental variables were used in the analysis but were eliminated as significant explanatory variables (see methods), these include: temperature, total dissolved N (TDN) concentration, dissolved organic N (DON) concentration, dissolved inorganic N (DIN) concentration, nitrate + nitrite (NOx) concentration, ammonium (NH4) concentration, Chlorophyll *a* concentration (Chla), salinity (Sal), turbidity (Turb), particulate organic C (POC), orthophosphate (PO4), stratification index (Supplementary Datatable 1; calculated using the equation of state for seawater density[1]). $\mathbf{R^2}$ = partial explained variation; $\mathbf{R^2}$ **Cumul.** and **Adj**$\mathbf{R^2}$ **Cumul.** = cumulative variance (non-adjusted and adjusted); **F-value** = Monte Carlo pseudo-F of a given variable; **P-value** = the original p-value; **P-value.adj** = the p-value adjusted for multiple testing using Bonferroni's correction.

**Term #**	**Variable**	$\mathbf{R^2}$	$\mathbf{R^2}$ **Cumul.**	**Adj**$\mathbf{R^2}$ **Cumul.**	**F-value**	**P-value**	**P-value.adj**
1	Temp	0.24	0.244	0.242	105.314	0.001	0.012
2	TDN	0.16	0.403	0.400	86.137	0.001	0.012
3	Chla	0.041	0.444	0.440	24.086	0.001	0.012
4	Sal	0.019	0.463	0.457	11.126	0.001	0.012

SUPPLEMENTARY MATERIAL REFERENCES

1. Millero, F. J. & Poisson, A. International one-atmosphere equation of state of seawater. *Deep Sea Res. Part A. Oceanogr. Res. Pap.* **28**, 625–629 (1981).
